# Differences in olfactory habituation between orthonasal and retronasal pathways

**DOI:** 10.1186/s12576-021-00822-0

**Published:** 2021-11-27

**Authors:** Wei Xiao, Zhifu Sun, Xiaoguang Yan, Xing Gao, Qianwen Lv, Yongxiang Wei

**Affiliations:** 1 Department of Otolaryngology Head & Neck Surgery, Zhongnan Hospital of Wuhan University, Wuhan, China; 2 Department of Otolaryngology Head & Neck Surgery, Beijing Anzhen Hospital, Capital Medical University, Beijing, China; 3 Smell and Taste Clinic, TU Dresden, Dresden, Germany; 4 Department of Otolaryngology Head & Neck Surgery, Capital Institute of Pediatrics, Beijing, China

**Keywords:** Orthonasal olfaction, Retronasal olfaction, Habituation, Phenethyl alcohol, *n*-Butyl acetate

## Abstract

The odorant arrives at nasal olfactory epithelium ortho- and retronasally. This experiment aimed to study the potential different olfactory habituation in orthonasal and retronasal pathways. 68 subjects were stimulated by constant airflow with an odor (50% phenethyl alcohol, PEA or 5% *n*-butyl acetate, BA) presented ortho- or retronasally. Participants rated the perceived odor intensity (0–10 points) per minute until the odor sensation disappeared. We also investigated the cross-habituation: when the subjects achieved full habituation, continue to rate odor intensity in a different pathway after instantly switching the odor stimulation pathway. The olfactory habituation curve was drawn. The differences of ratings between the orthonasal and retronasal olfaction at different time points and between male and female subjects were analyzed. The two odor intensity ratings decreased as the time extended, share the same “fast followed by slow” type. The ratings of orthonasal olfaction decreased faster than that of retronasal. The intensity rating of PEA of male retronasal approach was lower than that of female at the 5th min (*p* = 0.018). When orthonasal full habituation achieved, there was significant difference between the intensity ratings and the initial ratings of the retronasal stimulation pathway (*p* < 0.0001), and vice versa. We found obvious habituation as well as cross-habituation in both orthonasal and retronasal olfaction. The habituation of orthonasal olfaction was faster than that of retronasal olfaction. These different habituations were related to the gender.

## Background

The odorant molecules from the environment reach the olfactory epithelium through two different ways: orthonasal olfaction refers to the odorant molecules entering the nasal cavity when inhaling, and retronasal olfaction refers to the odorant molecules of food entering the nasal cavity through the nasopharynx when chewing and swallowing. The “Duality of Smell” hypothesis [1] considered that olfactory function as a dual system, but the potential mechanism is not clear. Aerodynamics studies have confirmed that the two different odor transport pathways do not interfere with each other [2]. In order to evaluate the retronasal olfaction, some scholars have studied the retronasal olfactory discrimination [3–5], and also studied the differences between the orthonasal and retronasal odor intensity evaluation [6], odor discrimination [7], and the relationship with oral movement [8].

In previous studies, a variety of experimental methods were used to achieve retronasal olfaction. For example, the odorant is dissolved in water and transported to the oral cavity [7, 9, 10]; a small container containing the odor solution is placed at the posterior part of tongue, and the odor is pumped out when the throat muscles contract [11, 12]. The subjects were trained to inhale through the mouth and exhale through the nose by using an odor stick attached to the hard palate [13] or conveying the air with the odor to the mouth through a silicone mouthpiece [14]. However, they are difficult to keep sustained and constant olfactory stimulation, accompanied by the interference of taste and mechanical stimulation, and the influence of abnormal physiological breathing mode on olfaction is difficult to evaluate. Small et al. [15] inserted two catheters into the nasal cavity to achieve orthonasal and retronasal olfaction. This method allows the subjects to join in the experiment in normal nasal breathing, and can achieve continuous and stable odor stimulation. However, the orthonasal olfaction of this method is unilateral nasal stimulation, the retronasal olfaction becomes bilateral. In order to get closer to the physiological state, we improved this method in our experiment.

Olfactory habituation is defined as a process that the decrease of olfactory sensitivity to the odor as the cause of repeated or prolonged exposure to a certain odor. For chefs, wine connoisseurs, perfume technicians, firemen or soldiers, the reduced odor sensitivity may cause adverse effects, or even serious consequences. Whether it can postpone the emergence of olfactory habituation or recover the sense of smell in time, which is very important for them.

However, our understanding of olfactory habituation is still very insufficient. In our study, the subjects were given continuous odor stimulation through the orthonasal and retronasal approaches. We carefully observed and analyzed the olfactory habituations of the two approaches, and then explored the potential neural mechanism of olfactory habituation.

## Materials and methods

### Subjects

The study was conducted according to the Declaration of Helsinki on Biomedical Research Involving Human Subjects and approved by the local ethics committee. Written informed consent was obtained from all subjects before the study.

A total of 68 healthy right-handed volunteers (32 men, 36 women; mean age, 28 years, range 23–35 years) participated in the study, who were assessed to have normal olfactory function by “Sniffin’ Sticks” test [16]. All participants had no history of medical, neurologic, psychiatric, or rhinal conditions, and were subjected to a detailed physical examination.

### Delivery apparatus

A catheter was inserted into the nasal cavity under the guidance of nasal endoscopy, one terminal of the catheter was end in the nasopharynx, the other terminal was end outside the anterior nostril, and the catheter was fixed on the nasal alar. The catheter was connected with a tube to deliver a constant airflow with odor to the nasopharynx, so as to realize the stimulation of retronasal olfaction, which is similar to the methods of Small et al. [15]. At the same time, the subjects wore a mask connected to the delivery tube to realize the orthonasal olfaction. Therefore, in our experiment, the subjects did not change the normal nasal breathing, and both the orthonasal and retronasal olfaction were bilateral. A small number of subjects reported slight discomfort during the operation, and the symptoms could be relieved after the catheter is fixed and a few minutes’ rest. We used a computer-controlled olfactometer (OG001; Burghart messtechnik, Wedel, Germany) to provide odor stimulation. When the clean airflow through the glass container with odorants in the olfactometer, the volatile molecules will be transported to the catheter which was in nasal cavity or mask worn by the subjects through the polytetrafluoroethylene pipeline. The airflow rate is constant (total flow, 2 l/min: relative humidity, 80%).

### Odorant stimulation

50% phenethyl alcohol (PEA) and 5% *n*-butyl acetate (BA) diluted in propylene glycol were chosen as odor stimulation. PEA smells like rose and is regarded as a pure olfactory stimulant, which does not stimulate trigeminal nerve; BA smells like banana, which can stimulate both olfactory nerve and trigeminal nerve [17, 18].

### Experimental protocol

All experiments were performed in the laboratory with well negative pressure ventilation. The subjects received continuous olfactory stimulation through orthonasal or retronasal approach. During the experiment, they sat comfortably in a chair, breathed steadily without sniffing. The visual analogue scale (VAS) was used to rate the odor intensity from 0 (hardly smell) to 10 (high intensity). When the subjects began to smell the odor (0 min), the initial ratings of the odors were rated. The researchers asked the subjects to rate the intensity every 1 min and recorded it until the subject reached sufficient habituation, that is, the odor could not be felt (the rating was 0), and the researchers recorded the total time. If the intensity rating did not decrease to 0 after 10 min, the stimulation was extended to 15 min, but only the ratings of the first 10 min were statistically analyzed. At this time, the researchers switched the olfactory stimulation approach (i.e., the orthonasal stimulation was converted to the retronasal stimulation, or the retronasal stimulation was converted to the orthonasal stimulation) and asked the subjects to immediately evaluate the intensity of the smell, so as to obtain the ratings of cross-habituation of different pathway.

The subjects participated in four experiments with the same procedure mentioned above. In order to minimize the possible cross-adaptation between odors and between the two pathways, the two odors were tested alternately with sufficient rest time. First, the retronasal olfactory stimulation experiment of PEA was carried out, followed after 5 min of rest, by the retronasal olfactory stimulation experiment of BA. After another 10 min of rest, the orthonasal olfactory stimulation experiment of PEA was carried out, followed after 5 min of rest, by the orthonasal olfactory stimulation of BA. This order of olfactory stimulation was to avoid possible background effects [19].

### Data analysis

Statistical analysis was performed, and the broken line charts and histograms were drawn using GraphPad Prism 6.0 (GraphPad Software, San Diego CA). The statistical significance between two groups was determined using Student’s *t*-test. Paired *t*-test was used to analyze the differences of olfactory intensity ratings at each time point between orthonasal and retronasal approach of all subjects, male or female subjects. Unpaired *t*-test was used to analyze the difference of initial intensity rating (0 min) between the two odors, the difference between the intensity rating of cross-habituation and the initial intensity rating of each odor, and the gender difference of the intensity rating of cross-habituation. Normally distributed variables were expressed as mean ± standard deviation (*x* ± *s*). Statistical significance was accepted at *p* < 0.05 (2-tailed tests).

## Results

During the orthonasal olfactory stimulation, all subjects could smell the odor when they inhaled, but not exhaled; on the contrary, during the retronasal olfactory stimulation, all subjects could smell the odor when they exhaled, but not inhaled. With the extension of stimulation, the two odor intensity ratings of different pathways decreased gradually. Among the 68 subjects, the intensity ratings of 10 subjects and 4 subjects did not drop to 0 after 15 min of continuous stimulation of orthonasally and retronasal PEA stimulation; the intensity score of 12 subjects 4 subjects did not drop to 0 after 15 min of continuous stimulation when they were exposed to orthonasal and retronasal BA. In order to standardize, the rating of first 10 min were counted.

The median duration to full olfactory habituation was 377.5 s for orthonasal PEA, 416 s for retronasal PEA, 358 s for orthonasal BA and 418 s for retronasal BA.

### Difference of initial intensity ratings between PEA and BA

For all subjects, the initial intensity ratings of the orthonasal and retronasal approaches of PEA were not significantly different (7.5 ± 0.3 and 8.0 ± 0.3, respectively, *p* = 0.064). The initial intensity ratings of the orthonasal and retronasal approaches of BA were not significantly different (8.1 ± 0.3 and 8.3 ± 0.3, respectively, *p* = 0.304). The initial intensity ratings of the orthonasal approach of PEA and BA were 7.5 ± 0.3 and 8.1 ± 0.3, respectively, and there was no significant difference between them (*p* = 0.088).

The initial intensity ratings of the retronasal approach of PEA and BA were 8.0 ± 0.3 and 8.3 ± 0.3, respectively, and there was no significant difference between them (*p* = 0.342).

### Olfactory habituation

**Fig. 1:**
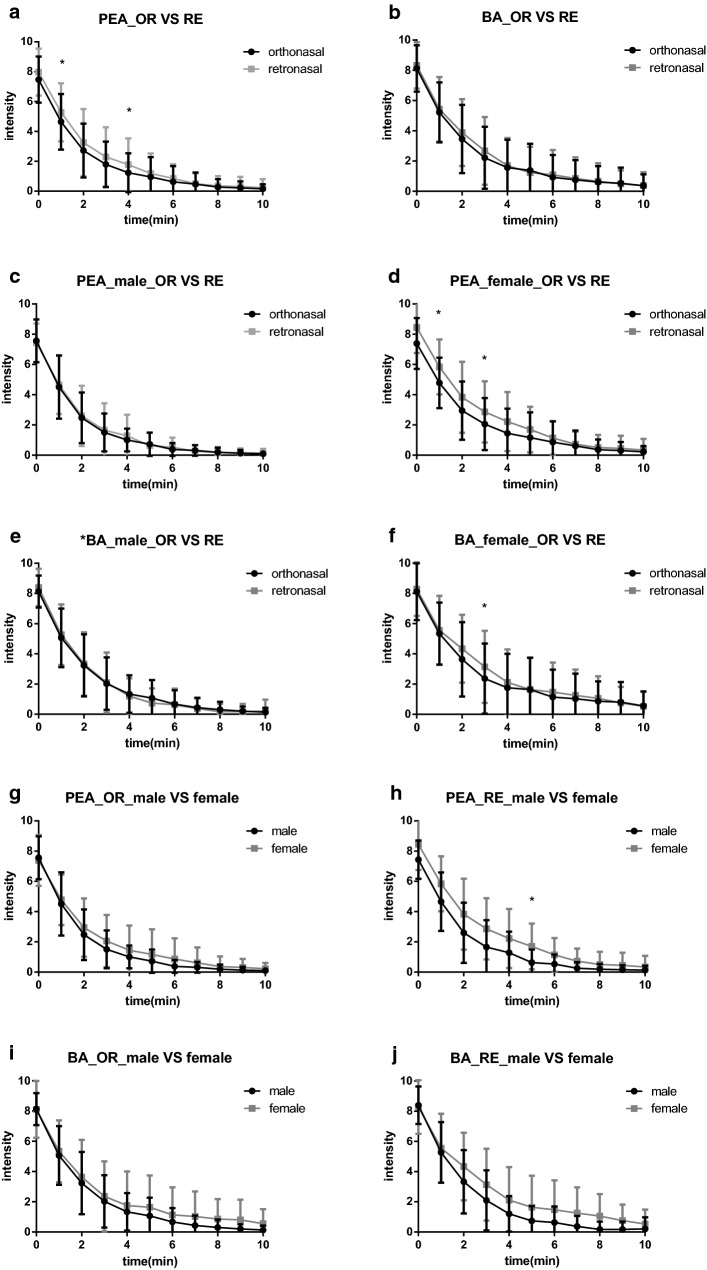
**a** Olfactory habituation of orthonasal and retronasal approaches to PEA in all subjects. **b** Olfactory habituation of orthonasal and retronasal approaches to BA in all subjects. **c** Olfactory habituation of orthonasal and retronasal approaches to PEA in male subjects. **d** Olfactory habituation of orthonasal and retronasal approaches to PEA in female subjects. **e** Olfactory habituation of orthonasal and retronasal approaches to BA in male subjects. **f** Olfactory habituation of orthonasal and retronasal approaches to BA in female subjects. **g** Gender differences in olfactory habituation of approach to PEA. **h** Gender differences in olfactory habituation of retronasal approach to PEA. **i** Gender differences in olfactory habituation of orthonasal approach to BA. **j** Gender differences in olfactory habituation of retronasal approach to BA

For all the subjects, the intensity ratings of orthonasal and retronasal approaches decreased significantly when they were continuously exposed to PEA or BA, and the intensity ratings of PEA of orthonasal approach decreased faster than that of retronasal approach, and the rating of the 1st min (4.6 ± 0.3; 5.3 ± 0.3) and the 4th min (1.2 ± 0.2; 1.8 ± 0.3) were statistically different (*p* = 0.029 and 0.038, respectively) (Fig. 1a). But there was no statistical difference in the ratings at each time point for BA (Fig. 1b).

When the male subjects were continuously exposed to PEA, there was no significant difference between the intensity ratings of orthonasal and retronasal approaches at each time point (Fig. 1c). But for female subjects, the ratings of the 1st min (4.8 ± 0.4; 5.8 ± 0.4) and the 3rd min (2.1 ± 0.4; 2.9 ± 0.5) were statistically different (*p* = 0.019 and 0.040, respectively) (Fig. 1d). When the male subjects were continuously exposed to BA, there was no significant difference between the intensity ratings of orthonasal and retronasal approaches at each time point (Fig. 1e). But for female subjects, the ratings of the 3rd min (2.4 ± 0.5; 3.1 ± 0.6) had statistical difference (*p* = 0.042) (Fig. 1f).

When continuously exposed to PEA, the intensity ratings of orthonasal approach of male subjects decreased slightly faster than that of female subjects, but there was no statistical difference at each time point (Fig. 1g). But for retronasal approach, there was significant difference in the rating of the 5th min (0.6 ± 0.2; 1.7 ± 0.4) (*p* = 0.018) (Fig. 1h). When continuously exposed to BA, the intensity ratings of orthonasal and retronasal approaches of male subjects decreased slightly faster than that of female subjects, but there was no significant difference in the scores at each time point (Fig. 1i, j).

### Cross-habituation

**Fig. 2:**
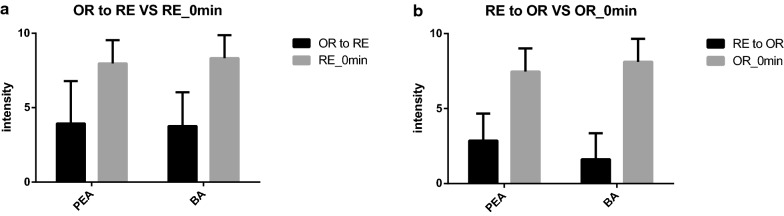
**a** Orthonasal to retronasal cross-habituation of PEA and BA. **b** Retronasal to orthonasal cross-habituation of PEA and BA

For all subjects, when the orthonasal PEA stimulation reached full olfactory habituation, the intensity rating of the retronasal approach was 3.9 ± 0.5, there were significant differences when compared with the initial intensity rating of the retronasal approach (8.0 ± 0.3) (*p* < 0.0001); when the orthonasal BA stimulation reached full olfactory habituation, the intensity rating of the retronasal approach was 3.8 ± 0.4, there are significant differences, when compared with the initial intensity rating of the retronasal approach (8.3 ± 0.3) (*p* < 0.0001) (Fig. 2a). There were also statistically significant differences for orthonasal PEA stimulation (2.9 ± 0.3; 7.6 ± 0.3, *p* < 0.0001) and orthonasal BA stimulation (1.6 ± 0.3; 8.1 ± 0.3, *p* < 0.0001), when the retronasal stimulations reached full olfactory habituation (Fig. 2b). There were significant differences between PEA and BA in the intensity ratings of orthonasal stimulation, when the retronasal stimulation reached full olfactory habituation (2.9 ± 0.3; 1.6 ± 0.3, *p* = 0.009). There was no significant difference between PEA and BA in the intensity ratings of retronasal stimulation, when the orthonasal stimulation reached full olfactory habituation (3.9 ± 0.5; 3.8 ± 0.4, *p* = 0.796).

## Discussion

According to Rankin [20], “habituation is defined as a behavioral response decrement that results from repeated stimulation and that does not involve sensory adaptation/sensory fatigue or motor fatigue.” The term adaptation has been used to describe neural processes (peripheral and cerebral) that constitute this decrease in behavioral response [21]. Most research on olfactory habituation focused on animal models, while the research on human olfactory habituation is relatively fewer. Thompson and Spencer identified nine behavioral characteristics of habituation in a landmark paper in the late 1960s [22]. In 2009, Rankin and his colleagues updated and revised description of the behavioral characteristics of habituation [20].

When one nostril has been preadapted to an odorant, the same odor presented to the same nostril or the other nostril without adaptation, it was rated less intense than before the adaptation, and there was a more significant drop in intensity rating when it was presented to the preadapted nostril [23], which proved that peripheral olfactory receptor neurons (ORNs) and central nervous system (CNS) were involved in olfactory adaptation. Studies have shown that olfactory adaptation can be affected by sniffing that can change the number of odor molecules reaching the olfactory epithelium [24]. The decline of electro-olfactogram (EOG) recorded during repeated olfactory stimulation was lower than that of subjective intensity ratings [25], but the adaptation of retronasal olfaction was not observed [26]. Animal experiments show that the piriform cortex acts as a powerful filter for continuous odor stimulation [27], and shows obvious adaptation [28], while almost no adaptation in olfactory bulb [29, 30]. In human functional magnetic resonance imaging (fMRI) studies, it was found that rapid adaptation occurred in the primary olfactory cortex (POC), especially in the piriform cortex [31]. During prolonged olfactory stimulation, the blood oxygenation level dependent (BOLD) signal in the hippocampus and anterior insular showed initial increase and then decrease, while the activity of orbitofrontal cortex was sustained [32, 33].

In our experiments, we observed significant habituation when subjects continuously exposed to PEA or BA through orthonasal and retronasal approaches. Previous studies on olfactory event-related potentials (OERPs) showed similar adaptive process of repetitive olfactory and trigeminal nerve stimulation [34, 35], and trigeminal nerve stimulation caused faster adaptation [35]. In our study, PEA was used as a pure olfactory stimulant, BA could stimulate both olfactory nerve and trigeminal nerve, which also proved that pure olfactory stimulation and also mixed stimulation with trigeminal nerve stimulation had obvious adaptation. Pierce et al. [14] did not observe the habituation of retronasal olfaction, it may be that the brain is always excited due to the sedulously trained breathing pattern of inhalation through the mouth and exhalation through the nose. At the same time, we observed that for all the subjects, the olfactory habituation speed of orthonasal was faster than that of retronasal when they were continuously exposed to PEA. When analyzing male and female subjects, respectively, we found that there was no significant difference in the ratings of orthonasal and retronasal olfactory intensity of male subjects at each time point, while the ratings of orthonasal olfactory intensity of female subjects decreased faster than that of retronasal. When analyzing the gender differences of olfactory habituation of two different stimulus approaches, we found that the olfactory habituation curves of male subjects were lower than that of female subjects, that is, male subjects had faster olfactory habituation than female subjects.

Gender differences in olfaction have always been a hot topic to researchers. Recent reports also suggested that women’s olfaction is superior to men's in threshold, discrimination and identification [36–38]. Our experiment first found that women’s ability to resist olfactory habituation is also better than men’s, that is, men are more likely to have olfactory habituation, and women can maintain relatively strong sensitivity when exposed to continuous odorant stimulation.

For both odors, we observed obvious cross-habituation, but not full habituation (i.e., intensity score > 0). This indicates that when the stimulus pathway changed, the olfactory system immediately sensed this change although it had been in the state of full habituation and recovered some sensitivity. It was shown that the orthonasal cross-habituation of BA was stronger than that of PEA when the retronasal stimulation reached full olfactory habituation, that is, trigeminal stimulation from the external world leads to stronger cross-habituation. The sensation of trigeminal nerve is related to pain and irritation, thus, the trigeminal responses would be larger than olfaction in the beginning, but when there are no harmful consequences after sensation, their value decreases rapidly [35].

We speculate that the sensitivity of olfactory receptor neurons gradually decreases when exposed to continuous odorant stimulation, but it cannot achieve full adaptation. The advanced olfactory center filters the continuous signals from the olfactory bulb, and finally the response of the cerebral cortex can be minimized. This is conducive to filtering the background or stable odorant, segmentation and component analysis of odorant mixture, so as to ignore the odor without vital importance and ensure that the brain is still responsive to new odor or odor changes. According to the different expression patterns of olfactory receptor genes in the mouse olfactory epithelium, bilateral nasal cavities can be symmetrically divided into a series of expression zones arranged along the dorsal–ventral and medial–lateral axes [39]. When olfactory stimulation is given through orthonasal or retronasal pathway, the time of odorant molecules in the airflow reaching each expression region will be slightly different. This regular arrangement of olfactory receptors may be one of the reasons for the difference of olfaction between the orthonasal and retronasal pathways. Moreover, when olfactory receptor neurons project to the olfactory bulb, the neurons expressing the same receptor project to specific subset of glomeruli, giving rise to a stereotyped and highly organized information map [40, 41]. Therefore, when switching to another stimulus pathway at the time of full adaptation, the neural signals generated by the partially adaptive olfactory receptor neurons are weak, and this information reaches the olfactory cortex after different temporal and spatial coding in olfactory bulb. This kind of differently coded information can wake up part of the sensitivity of the olfactory cortex, giving rise to incomplete cross-habituation in behavioral performance.

## Conclusion

We found obvious habituation and the characteristics of “fast followed by slow” in both orthonasal and retronasal olfaction. The orthonasal habituation was faster than that of orthonasal. This difference is shown in female subjects, while there is no difference in male subjects. Retronasal olfaction of male was more likely to habituate than female. There is obvious cross-habituation between orthonasal and retronasal approaches, but not full adaptation.

## Data Availability

Data could be obtained upon request to the corresponding author.

## Abbreviations

PEA: Phenethyl alcohol
BA: *n*-Butyl acetate
VAS: Visual analogue scale
ORNs: Olfactory receptor neurons
CNS: Central nervous system
EOG: Electro-olfactogram
fMRI: Functional magnetic resonance imaging
POC: Primary olfactory cortex
BOLD: Blood oxygenation level dependent
OERPs: Olfactory event-related potentials

## References

[1] Rozin P (1982). “Taste-smell confusions” and the duality of the olfactory sense. Atten Percept Psychophys.

[2] Ni R, Michalski MH, Brown E (2015). Optimal directional volatile transport in retronasal olfaction. Proc Natl Acad Sci USA.

[3] Heilmann S, Strehle G, Rosenheim K, Damm M, Hummel T (2002). Clinical assessment of retronasal olfactory function. Arch Otolaryngol Head Neck Surg.

[4] Haxel BR, Bertz-Duffy S, Faldum A (2011). The candy smell test in clinical routine. Am J Rhinol Allergy.

[5] Renner B, Mueller CA, Dreier J, Faulhaber S, Rascher W, Kobal G (2009). The candy smell test: a new test for retronasal olfactory performance. Laryngoscope.

[6] Heilmann S, Hummel T (2004). A new method for comparing orthonasal and retronasal olfaction. Behav Neurosci.

[7] Hannum M, Stegman MA, Fryer JA, Simons CT (2018). Different olfactory percepts evoked by orthonasal and retronasal odorant delivery. Chem Senses.

[8] Burdach KJ, Doty RL (1987). The effects of mouth movements, swallowing, and spitting on retronasal odor perception. Physiol Behav.

[9] Cerf-Ducastel B, Murphy C (2001). fMRI activation in response to odorants orally delivered in aqueous solutions. Chem Senses.

[10] Diaz ME (2004). Comparison between orthonasal and retronasal flavour perception at different concentrations. Flavour Fragr J.

[11] Sun BC, Halpern BP (2005). Identification of air phase retronasal and orthonasal odorant pairs. Chem Senses.

[12] Lee J, Halpern BP (2013). High-resolution time–intensity tracking of sustained human orthonasal and retronasal smelling during natural breathing. Chemosens Percept.

[13] Pellegrino R, Atchley A, Ali S, Shingleton J, Luckett CR. Retronasal habituation: characterization and impact on flavor perception using time-intensity. bioRxiv. 2018.

[14] Pierce AM, Simons CT (2018). Olfactory adaptation is dependent on route of delivery. Chem Senses.

[15] Small DM, Gerber JC, Mak YE, Hummel T (2005). Differential neural responses evoked by orthonasal versus retronasal odorant perception in humans. Neuron.

[16] Hummel T, Sekinger B, Wolf SR, Pauli E, Kobal G (1997). 'Sniffin' sticks': olfactory performance assessed by the combined testing of odor identification, odor discrimination and olfactory threshold. Chem Senses.

[17] Doty RL, Brugger WE, Jurs PC, Orndorff MA, Snyder PJ, Lowry LD (1978). Intranasal trigeminal stimulation from odorous volatiles: psychometric responses from anosmic and normal humans. Physiol Behav.

[18] Wang J, Rupprecht S, Sun X (2017). A free-breathing fMRI method to study human olfactory function. J Vis Exp.

[19] Schifferstein H (1996). Cognitive factors affecting taste intensity judgments. Food Qual Preference.

[20] Rankin CH, Abrams T, Barry RJ (2009). Habituation revisited: an updated and revised description of the behavioral characteristics of habituation. Neurobiol Learn Mem.

[21] Pellegrino R, Sinding C, de Wijk RA, Hummel T (2017). Habituation and adaptation to odors in humans. Physiol Behav.

[22] Thompson RF, Spencer WA (1966). Habituation: a model phenomenon for the study of neuronal substrates of behavior. Psychol Rev.

[23] Zhou W, Chen D (2009). Binaral rivalry between the nostrils and in the cortex. Curr Biol.

[24] Beauchamp J, Scheibe M, Hummel T, Buettner A (2014). Intranasal odorant concentrations in relation to sniff behavior. Chem Biodivers.

[25] Hummel T, Knecht M, Kobal G (1996). Peripherally obtained electrophysiological responses to olfactory stimulation in man: electro-olfactograms exhibit a smaller degree of desensitization compared with subjective intensity estimates. Brain Res.

[26] Hummel T, Seo HS, Pellegrino R (2017). Electro-olfactograms in humans in response to ortho-and retronasal chemosensory stimulation. Chemosens Percept.

[27] Kadohisa M, Wilson DA (2006). Olfactory cortical adaptation facilitates detection of odors against background. J Neurophysiol.

[28] Wilson DA (1998). Habituation of odor responses in the rat anterior piriform cortex. J Neurophysiol.

[29] Zhao F, Holahan MA, Houghton AK (2015). Functional imaging of olfaction by CBV fMRI in monkeys: insight into the role of olfactory bulb in habituation. Neuroimage.

[30] Zhao F, Wang X, Zariwala HA (2016). fMRI study of olfaction in the olfactory bulb and high olfactory structures of rats: Insight into their roles in habituation. Neuroimage.

[31] Sobel N, Prabhakaran V, Zhao Z (2000). Time course of odorant-induced activation in the human primary olfactory cortex. J Neurophysiol.

[32] Li W, Luxenberg E, Parrish T, Gottfried JA (2006). Learning to smell the roses: experience-dependent neural plasticity in human piriform and orbitofrontal cortices. Neuron.

[33] Poellinger A, Thomas R, Lio P (2001). Activation and habituation in olfaction—an fMRI study. Neuroimage.

[34] Scheibe M, Opatz O, Hummel T (2009). Are there sex-related differences in responses to repetitive olfactory/trigeminal stimuli. Eur Arch Otorhinolaryngol.

[35] Flohr EL, Boesveldt S, Haehner A, Iannilli E, Sinding C, Hummel T (2015). Time-course of trigeminal versus olfactory stimulation: evidence from chemosensory evoked potentials. Int J Psychophysiol.

[36] Sorokowski P, Karwowski M, Misiak M (2019). Sex differences in human olfaction: a meta-analysis. Front Psychol.

[37] Oleszkiewicz A, Schriever VA, Croy I, Hähner A, Hummel T (2019). Updated Sniffin’ Sticks normative data based on an extended sample of 9139 subjects. Eur Arch Oto-Rhino-Laryngol.

[38] Hummel T, Kobal G, Gudziol H (2007). Normative data for the “Sniffin' Sticks” including tests of odor identification, odor discrimination, and olfactory thresholds: an upgrade based on a group of more than 3,000 subjects. Eur Arch Otorhinolaryngol.

[39] Ressler KJ, Sullivan SL, Buck LB (1993). A zonal organization of odorant receptor gene expression in the olfactory epithelium. Cell.

[40] Ressler KJ, Sullivan SL, Buck LB (1994). Information coding in the olfactory system: evidence for a stereotyped and highly organized epitope map in the olfactory bulb. Cell.

[41] Sullivan SL, Bohm S, Ressler KJ, Horowitz LF, Buck LB (1995). Target-independent pattern specification in the olfactory epithelium. Neuron.

